# Eosinophilic Gastroenteritis: Case Report and Review in Search for Diagnostic Key Points

**DOI:** 10.1155/2015/239506

**Published:** 2015-05-05

**Authors:** Guillermo López-Medina, Manuel Gallo, Alejandro Prado, Iliana Vicuña-Honorato, Roxana Castillo Díaz de León

**Affiliations:** ^1^Hospital Angeles Clinica Londres, Durango No. 50, Roma Norte, Cuauhtémoc, 06700 Ciudad de México, DF, Mexico; ^2^Hospital Angeles Mocel, Gregorio V. Gelati No. 29, San Miguel Chapultepec, Miguel Hidalgo, 11850 Ciudad de México, DF, Mexico

## Abstract

Eosinophilic gastroenteritis is considered an uncommon disease with a low incidence rate that remains as a diagnostic challenge for the clinician, in spite of the fact that seventy years have passed since its original description. Hereby we present the case of a 29-year-old male without history of allergies who was evaluated for unspecific gastrointestinal symptoms, without relevant findings on physical examination and presenting an initial complete blood count (CBC) with severe eosinophilia. The patient was evaluated and the diagnosis of eosinophilic gastroenteritis was confirmed by histopathological findings. The relevance of the case resides in highlighting the lack of guidelines or consensus for histological diagnosis being virtually the only one available. To a similar extent, treatment evidence is based on case series with a reasonable number of patients and case reports.

## 1. Introduction

Primary eosinophilic gastrointestinal diseases include five variants according to their localization on the gastrointestinal tract: esophagitis, gastritis, gastroenteritis, enteritis, and eosinophilic colitis. As to gastroenteritis, stomach and small bowel are the most affected segments by 26% to 81% and 28% to 100%, respectively, often associated with simultaneous infiltration of the esophagus, colon, and rectum with a minor intensity. Reports of localized infiltration to the biliary tract come to emphasize that clinical spectrum may vary to a large extent, in accordance with the affected site and depth of the infiltration [1–4].

## 2. Case Description

**Figure 1:**
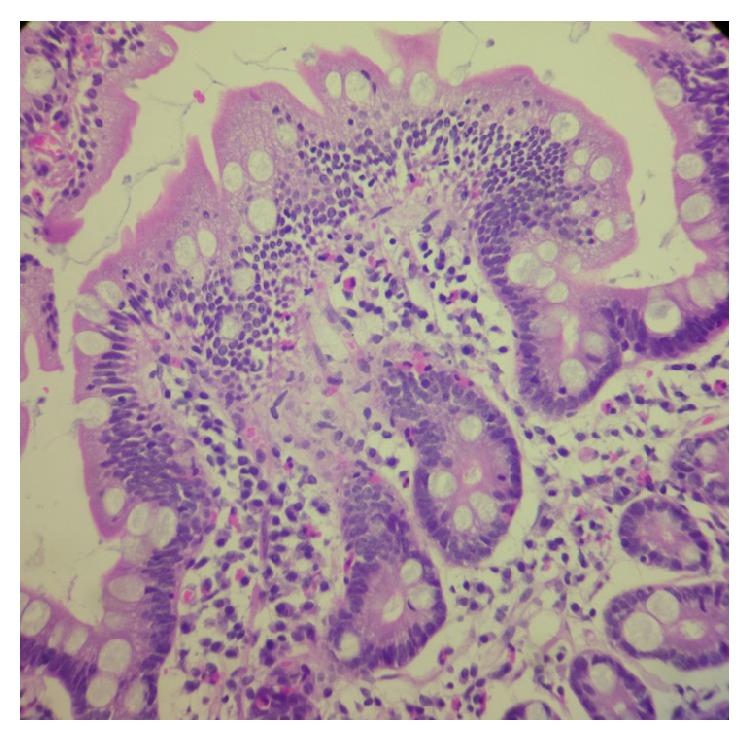
Photomicrography of a duodenal mucosal biopsy specimen (40x) shows a villous surface with hypercellular lamina propria and eosinophilic infiltrates.

**Figure 2:**
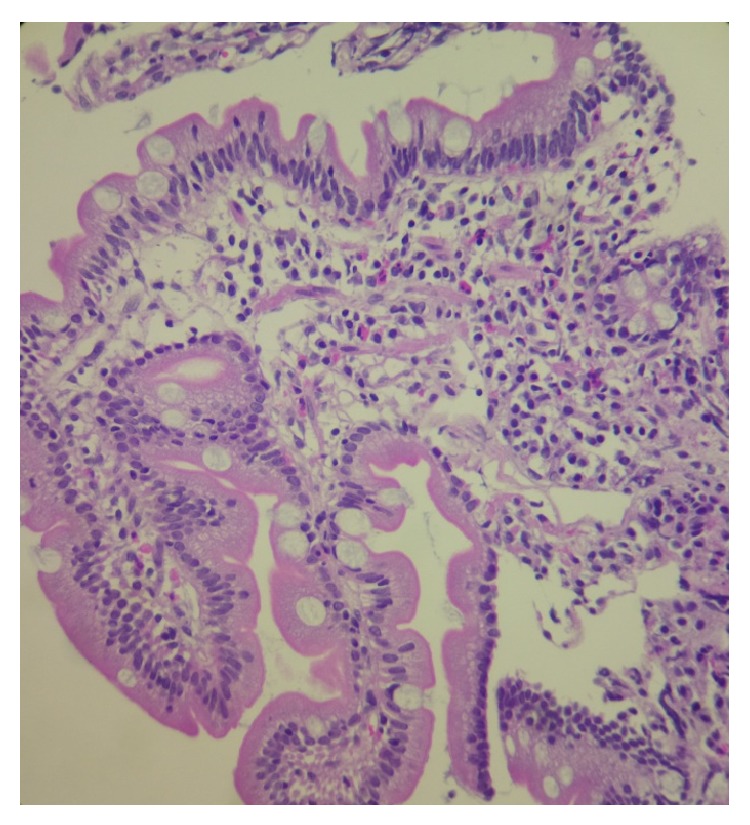
Photomicrography of a duodenal mucosal biopsy specimen (40x) depicts predominant eosinophilic inflammation within the lamina propria, and the glands appeared normal.

**Figure 3:**
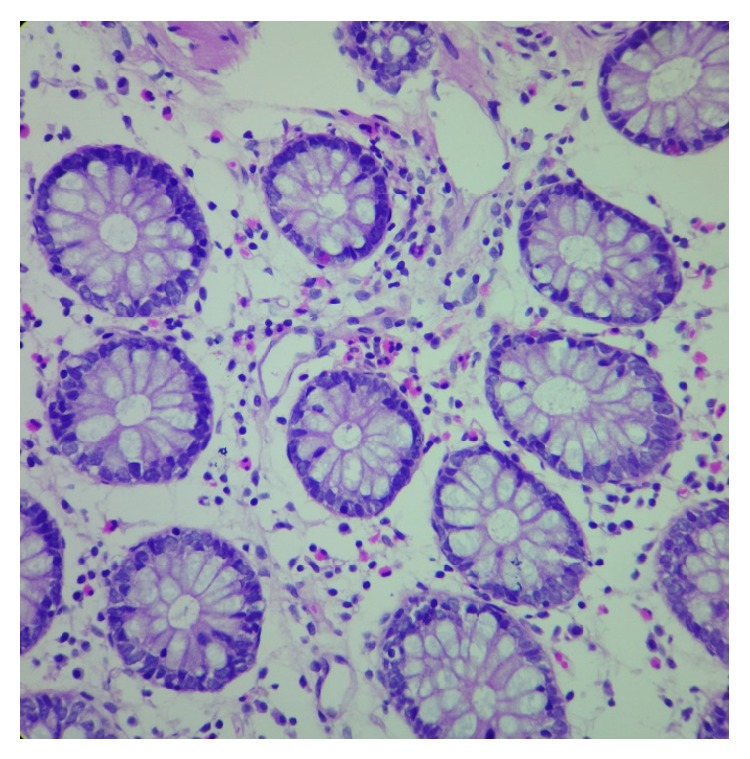
Photomicrography of a colonic mucosal biopsy specimen (40x) with chronic lymphoid inflammation, accentuating the gross number of eosinophils: the glands appeared normal.

**Figure 4:**
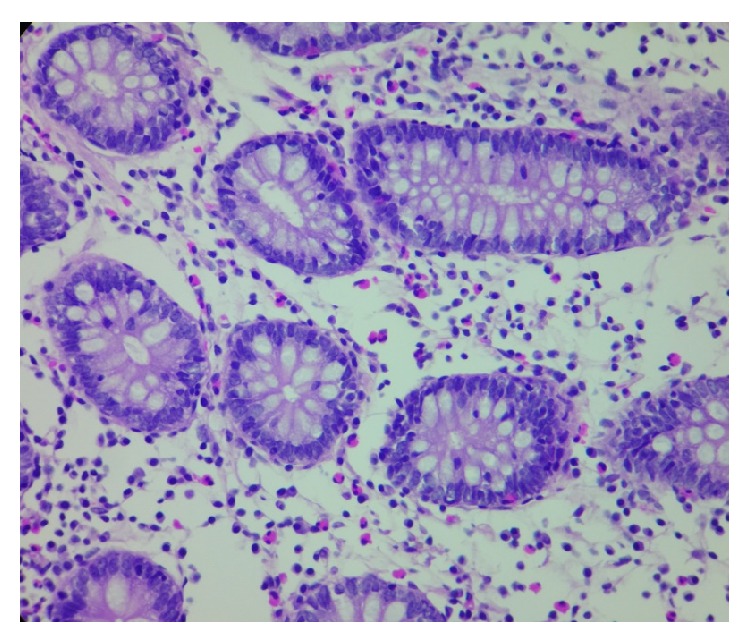
Photomicrography of a colonic mucosal biopsy specimen (40x) showing oedema of the lamina propria and chronic inflammatory infiltrate along with abundant eosinophils.

We present a 29-year-old male, resident of an urban area, single, and without relevant family background, who consumes alcoholic beverages once a weak, denies smoking, allergies or previous transfusions. History of acute viral hepatitis type A during his childhood, pneumococcal, and flu vaccines were applied two weeks prior to his admission. Surgical history of amygdalectomy, appendectomy, renal lithotomy, and installation of double J stent secondary to renal lithiasis, all without noticed complications. He arrived to the emergency department with a chief complaint of three days prior to his admission with liquid depositions without blood or mucus, in 10 occasions approximately, along with vomit of gastric content. Within the next 48 hrs, moderate intensity, acute epigastric pain appeared. Fever was not reported. At the initial physical examination, he presented stable vital signs with palpable cervical lymph nodes, which were painless, mobile, without features suggesting malignancy and abdominal pain were discovered at palpation of the inferior quadrants, rest of the examination ended without further findings. The CBC resulted in a white blood cell count of 13.1 × 10^3^/mm^3^ and 3,537/mm^3^ (27%) corresponding to eosinophils, and the rest of cell count was within normal range. Parasitoscopic fecal analysis was positive for* Entamoeba histolytica* trophozoites and cysts, metronidazole plus iodoquinol were initiated. The parasitic infestation did not explain the severe eosinophilia; therefore, an abdominal ultrasonogram was performed and evidenced free fluid in both iliac fossae and pelvis, and an image compatible with a calculus within the inferior pole of left kidney was also observed. Abdominal fluid was obtained via an ultrasound guided puncture resulting in albumin of 1.7 d/dL, proteins 3.3 gr/dL, glucose 65 mg/dL, total cells of 4400/mm^3^, 70% corresponding to eosinophils, and cultures tested negative for bacterial or fungi growth. Following the assessment of the ascites a contrast computed tomography confirmed free fluid in the right parietocolic gutter from epigastrium to pelvis, and a calculus on the inferior pole of the left kidney and thickening of the wall of the terminal ileum, enlarged pelvic, and inguinal lymph nodes were observed as well. Under the suspicion of intestinal malignancy a superior endoscopy revealed a redundant esophagus with generalized, moderate epithelium thickening, diffuse, moderate inflammatory process of the gastric mucous, normal antrum, and pylorus and duodenum, and urease test was negative for* Helicobacter pylori*. Samples from the esophagus and stomach were not taken. Colonoscopy reported normal perianal structures, spastic and redundant rectumsigmoid with normal mucosa, and the rest of colon without alterations. Biopsies of duodenum and colon were taken and the histopathologic study concluded unspecific mild chronic duodenitis without atrophy of vellus and moderate eosinophilia (mean 30 eosinophils/high power field), and no microorganisms by routine tinctions nor histopathological findings suggestive of intestinal malabsorption were identified (Figures 1 and 2) and mild chronic colitis with moderate eosinophilia (mean of 24 eosinophils/high power field) for the colon specimens (Figures 3 and 4). Microscope slides from both biopsies were revised with the maximum magnification (40x) in the form of “sweeping,” that is, downwards then upwards and from left to right. Because of the small sized samples and the limited amount of tissue available, a “skipped” overview was not considered an option. The number of eosinophils in each field was counted and the total was divided by the absolute number of fields, in this way obtaining an average number of eosinophils. Serum IgE concentration was normal. Given the histopathological findings, ascites with a marked eosinophil predominance in the absence of an infectious or neoplastic disease, supported by high serum eosinophil concentration, the diagnosis of eosinophilic gastroenteritis was concluded. While no attributed allergenic source was evident, he received inpatient treatment base on a gluten and dairy free diet, minimum seasoning, and well-cooked poultry and beef, without administration of steroids, being effective by inducing remission in this patient. At the time of discharge the patient persisted with an elevated count of eosinophils of 3,002/mm^3^. A couple of months after discharge under the same dietary regimen, the patient showed sustained clinical remission.

## 3. Discussion

Eosinophilic gastroenteritis is a nonfrequent primary gastrointestinal disease of which its etiology is not fully understood and characterized by histopathologic eosinophilic infiltrates in one or more segments from de stomach to the rectum [5]. Since its initial description by Kaijser in 1937 reports has shown that it may affect adults as well as pediatric population, preponderance within the male gender has been suggested [6]. It is important to differentiate it from secondary diseases associated with eosinophilia of which eosinophilic accumulation has an identifiable cause, like hypereosinophilic syndrome, inflammatory intestinal diseases, infections particularly by helminthes, vasculitis such as Churg-Strauss or polyarteritis nodosa, connective tissue diseases such as systemic lupus erythematosus, scleroderma, dermatomyositis, neoplasms, graft versus host disease in bone marrow transplant recipient patients, secondary reactions to nonsteroidal anti-inflammatory drugs, interferon, enalapril, carbamazepine, trimethoprim/sulfamethoxazole, clopidogrel, food allergies,* Helicobacter Pylori* infection, all of the above considered as differential diagnosis [7, 8].

The estimated prevalence reported for eosinophilic gastroenteritis is 1/100 000; however given the rarity of the diagnosis it is yet underestimated [5]. Six series have been published with a significant number of cases worldwide, and Talley et al. gathered 40 cases during a period from 1950 to 1986, Chang et al. gathered 59 new cases from 1987 to 2010, de Chambrun et al. presented 49 cases from 1988 to 2009, and Chen et al. from 1984 to 2002 described 15 patients, just to name a few [5, 9], highlighting the scarcity of this entity.

All ethnic groups may be affected, between 20 and 50 years of age, commonly around the third decade of life, 70% of the cases have personal and/or family history of allergies such as atopia, eczema, asthma, or allergic rhinitis. Unlike eosinophilic colitis most patients with gastroenteritis variant present elevated total serum IgE concentration [2, 8, 10].

The pathophysiology of eosinophilic gastroenteritis remains not fully comprehended. A hypersensitivity response is strongly suggested by clinical improvement reported in patients managed with corticosteroids. As part of the innate defense system, the presence of eosinophils in the intestinal lamina propria is a normal finding; however infiltration to deeper layers is considered abnormal. It is known that peripheral eosinophil concentration greater than 1.5 × 10^9^/L can produce tissular damage regardless of the underlying cause [11, 12]. The main cytokines involved in the pathogenesis of this condition are IL-3, IL-5, granulocytes, and macrophages colony stimulating factors (GM-CSF); IL-5 plays a major role being the most potent, selective, chemotactic factor along with eotaxin (CCL11) for the migration of eosinophils towards the intestinal mucosa, promoting degranulation and inhibiting their apoptosis, remaining highly activated for any event that alters the intestinal mucosa, even in noninflammatory states [4, 11].

Eosinophils dwelling within the intestinal mucosa participate in the innate immune response mainly against helminthic infections and those caused by Mycobacteria spp.,* Isospora belli*,* Sarcocystis coccidiomycosis*,* Dientamoeba fragilis*, and HIV infection; hence, gastrointestinal eosinophilia is usually not associated with infections of common bacteria, viruses, or fungus. Other agents like intestinal protozoa including giardiasis and amebiasis do not course with eosinophilia [4, 13].

Clinical manifestations thoroughly vary depending on their location within the gastrointestinal tract and depth through the intestinal wall; dysphagia, abdominal pain, and diarrhea are the most frequent symptoms in adults, and other symptoms are stenosis, hemorrhage, ulcers, and a wide range of motility alterations. Up to 80% manifest symptoms for several years, rarely presenting as an acute abdomen or intestinal perforation, all of the above should raise suspicion of a tumor which must be ruled out [10, 11].

The classification proposed in 1970 by Klein et al. has been the most employed, based on the depth of the eosinophilic infiltration; it can be divided according to mucous, muscular o serous involvement [2]. The mucous variant is the most common ranging from 25% to 100%, however this is not reliable due to diagnosis bias, being serous samples the least obtained for histopathological assessment. The key manifestations in mucous variant include protein-losing enteropathy, bleeding or malabsorption, nausea, vomit, diarrhea, and dyspepsia that do not respond to antisecretory therapy; the above symptoms can be confused with irritable bowel syndrome, pancreatitis, dyspepsia, appendicitis, or inflammatory intestinal disease. Muscular affection comprehends 13% to 70% of cases, manifested as thickening of the wall and obstructive symptoms such as colic pain, nausea, and vomit; it is rare to find true stenosis and, if present, jejunum is the most common site and less frequent as a cecal mass. Affection of the serosa exists in 14% to 40%, and ascites with predominance of eosinophils are the typical manifestation, along with abdominal distention and a large peripheral blood count of eosinophils, characteristically with good response to corticosteroid therapy [2, 11].

The initial workup comprehends a complete medical history and exhaustive physical examination, a CBC and chemistry panel. The hemogram will typically show eosinophilia in 20% to 80% of the cases, with a mean serum count of 2000 eos/*μ*L when the mucosa is involved, 1000 eos/*μ*L and 8000 eos/*μ*L for the muscular wall, and the serosa, respectively. It is possible to find sideropenic anemia and hypoalbuminemia especially in association with the mucous variant and a case has been reported of hypercholesterolemia as a manifestation of gastroenteritis within the duodenal muscularis mucosae as part of a protein-losing enteropathy [11, 14]. The production of eosinophils is moderated by a net of cytokines that maintain their normal peripheral blood count between 0.05 and 0.5 × 10^9^/L and their concentration in bone marrow aspiration between 1% and 6%. It is not normal to find eosinophils in the rest of the human economy, except for the thymus, spleen, lymphatic nodules, uterus, and gastrointestinal tract from stomach to rectum as mentioned above; however, it is important to notice that the normal count has not been yet determined [15]. Efforts have been made into establishing the normal eosinophil count throughout different segments of the gastrointestinal tract; nevertheless, it may not be widespread since the evidence is limited to small samples and including just a few ethnic groups in the same way as for pediatric population [16, 17].

According to the “2011 Year Working Conference on Eosinophil Disorders and Syndromes” the term hypereosinophilia is defined as a blood count of eosinophils greater than 1.5 × 10^9^/L in two samples, taken 1 month apart and/or hypereosinophilia in tissues defined by (1) eosinophils greater than 20% of all nucleated cell in bone marrow and/or (2) eosinophilic infiltration cataloged as extensive according to pathologist opinion and/or (3) marked deposition of protein granules of eosinophils in presence or absence of important tissue infiltration. Hypereosinophilia must fulfill the above criteria plus evidence of specific organ damage. However eosinophilic gastroenteritis is excluded and categorized as an organ-restricted condition accompanied by hypereosinophilia, reason by which no definitive criteria have been determined for this entity [15]; therefore peripheral eosinophilia is not an universal phenomenon in the context of eosinophilic gastroenteritis [11, 15].

The stool analysis will show up to 30% of patients, mild to moderate steatorrhea. In relation to image studies, nodular or irregular thickening of the stomach or small bowel folds can be found on computed tomography [11]. Ultrasonogram is useful in the search for ascites in the variation affecting the serosa and guided paracentesis will result in being sterile with eosinophilic cellular predominance. Endoscopy is poorly specific showing a friable mucosa, nodular changes or ulcers, occasionally diffuse inflammation with vellus atrophy, submucosa oedema, or fibrosis. Technetium (99mc) exametazime labeled leukocytes single photon emission computed tomography (SPECT/CT) can be useful for evaluating extension but not for establishing the diagnosis [8, 11].

In the histopathological study of 6 samples from normal and abnormal areas, the diagnosis is suggested by the presence of eosinophilic infiltrates in intestinal crypts of gastric glands, extracellular deposition of eosinophilic remnants such as mayor basic protein or cationic protein, and incremented infiltration by mastocytes, keeping in mind that even the processing of samples may activate eosinophil degranulation [8, 10, 11]. For the muscular variant full thickness biopsy is required via laparoscopy since endoscopic biopsy is useless in the absence of compromise of the mucosa. The infiltration of the colon may be diffuse affecting the lamina propria with focal aggregates reaching the muscularis mucosae preserving the underlying submucosa [8].

In sum, in order to reach a diagnosis the following are required: (1) gastrointestinal symptoms, (2) eosinophilic infiltration in one or more segments of the gastrointestinal tract, by measuring the number of eosinophils under high power field view, without established threshold ranging from more than 20 to even more than 50 eosinophils, and (3) exclusion of other causes that course with eosinophilic intestinal infiltration [11, 18, 19].

Once the diagnosis of gastroenteritis is established, an evaluation by an allergologist is helpful in practicing environmental allergen detection, food allergy testing, food specific IgE by immuno-CAP and atopy patch test (APT), since triggers mediated by IgE make diet modifications poorly effective [8].

The first step of treatment includes withdraw of common allergens in the diet; nevertheless the therapeutic response is highly variable. The treatment with steroids shows an improvement in up to 90% of cases; however, the duration is not specified, and relapse is not uncommon; hence, treatment tends to extend. There is no consensus about the optimal type or dose of steroid; however, budesonide has the advantage of a local effect and a first step metabolism which entails less risk for adrenal suppression; prednisolone at 20 to 40 mg/day, for 6 to 8 weeks including the tapering, has been the most utilized regimen. Sodium cromoglicate, ketotifen, and montelukast have been proposed as therapeutic measures with inconclusive results. Surgery becomes useful in cases of obstructive symptoms lacking improvement with medical treatment. Parental nutrition will be useful in cases where patient comorbidities exclude the enteral route [11, 18, 19].

Not much is known about the natural evolution and prognosis of this disease, and it is possible that different segments are affected in the course of time or even progress to a complete hypereosinophilic syndrome which entails extraintestinal involvement for which endoscopic and cardiopulmonary follow-up is recommended [8].

## 4. Conclusions

Since its initial description more than 70 years ago, the efforts for characterizing the pathophysiology process and establishing standard diagnostic criteria for eosinophilic gastroenteritis have been scarce. A nondespicable number of cases have been reported, which indicates that clinical suspicion is increasing despite its low incidence.

This entity emphasizes the importance of an invasive approach along with a thorough medical history; findings on physical examination are not useful for reaching the diagnosis, hence being fundamentally histological.

Unlike other forms of primary eosinophilic gastrointestinal diseases such as eosinophilic esophagitis, there are no clinical guidelines available for eosinophilic gastroenteritis; therefore, it will remain underdiagnosed, with uncertain prognosis, and as for the patient we can only offer low evidence-based therapeutic options.
